# Learning With Virtual Reality in Nursing Education: Qualitative Interview Study Among Nursing Students Using the Unified Theory of Acceptance and Use of Technology Model

**DOI:** 10.2196/20249

**Published:** 2020-09-01

**Authors:** Ann-Kathrin Lange, Jana Koch, Anastasia Beck, Till Neugebauer, Frauke Watzema, Kamil J Wrona, Christoph Dockweiler

**Affiliations:** 1 University of Bielefeld Bielefeld Germany

**Keywords:** virtual reality, edutainment, serious games, education, health care, gamification, anatomy, digital game–based learning, nursing, nursing informatics

## Abstract

**Background:**

Digital games–based learning is a method of using digital games to impart knowledge. Virtual reality (VR) programs are a practical application of this method. Due to demographic changes, the nursing profession will become increasingly important. These VR applications can be of use in training nurses for future professional challenges they may encounter. The continuous development of VR applications enables trainees to encounter simulated real life effectively and to experience increasingly concrete situations. This can be of great importance in nursing education, since 3-dimensionality enables a better visualization of many fields of activity and can prevent potential future errors. In addition to this learning effect, VR applications also bring an element of fun to learning.

**Objective:**

The aim of this qualitative research effort is to observe the degree of acceptance of VR applications by nursing students in Germany. Various factors, including social influences, performance expectations, and effort expectations, are taken into consideration.

**Methods:**

With a qualitative cohort study, the acceptance of nursing students towards VR applications in anatomy teaching was determined. The 12 participants were first asked to fill out a quantitative questionnaire on their sociodemographic characteristics and the extent to which they valued and liked using technology. The participants were then allowed to test the VR application themselves and were finally asked about their experience in a qualitative interview. For the collection of data and the analysis of results, the unified theory of acceptance and use of technology was used in this study.

**Results:**

Overall, the study shows that the interviewed persons rated the VR application quite positively. The greatest influence in this was the personal attitude towards technology; the higher this affinity is, the more useful the VR application appears. Social influences can also increase the participant’s own acceptance if peers have a positive attitude towards such applications. The study shows that the trainees' motivation to learn was increased by using VR. We believe this is because each trainee could learn individually and the VR application was perceived as an enjoyable activity. Nevertheless, the cost factor of implementing VR applications in nursing training is currently still an obstacle, as not every institution has such financial capacities.

**Conclusions:**

The extent to which the use of VR applications in the training of nursing staff is justified depends on the degree of personal acceptance. The collected results give good practice-oriented insight into the attitude of trainees towards VR. Many of the interviewed persons saw benefits in the use of VR technologies. As VR applications are constantly developing, it is necessary to conduct further studies on VR applications in nursing education and to include other possible disciplines in which these applications can be helpful.

## Introduction

Nursing or health care support staff work mainly in hospitals, outpatient facilities, and (partially) inpatient facilities [1]. The conditions of the profession and training are regulated by law, as well as the terms “nurses” and “health care professionals” themselves, who are responsible for general care. The vocational training usually takes about 3 years and consists of theoretical as well as practical content, concluding with a state exam [2]. However, the training places have some leeway in regard to the implementation of the external conditions [3]. The 2017 Nursing Professions Act (*Pflegeberufe Reformgesetz*) called for a merger of the nursing care law (*Krankenpflegegesetz*) with the geriatric care law (*Altenpflegegesetz*) from 2020 onwards [4]. Geriatric care, nursing care, and pediatric care are to be part of a generalized apprenticeship and nurses are to be asserted as care experts [4]. Nowadays, the work density, growing bureaucracy, working time regulations, and many other factors increase the burden on nurses [5]. It is difficult to recruit nursing trainees [6]. In addition, the demographic change intensifies this situation. Due to a change in the age structure in society, the amount of elderly people in proportion to young people is increasing. Because illnesses are associated with age, an increase of chronic and multimorbid illnesses is to be expected, while at the same time a decline of the working population takes place [7]. There is also a higher need for qualified nurses, which is partially being covered by untrained staff [1]. In order to prepare future nurses for oncoming challenges, it is important to have a qualitative apprenticeship. Content should be as optimized as possible and be passed on to a wide range of students. It is also important to offer attractive terms and conditions in order to win new trainees.

One possibility to do so is by integrating games into the learning process. As Zupanic et al [8] discovered, almost every trainee in the health care sector uses electronic learning. Therefore, there are different concepts regarding the transfer of information through playful elements. This way, the motivation to learn increases and the player is stimulated [9,10]. The primary function of gaming is not information transfer but influencing thought and action [11]. When taking into consideration which of the current teaching and learning methods are attractive, it is important to note the progress in society as well as in technology. A suitable principle is digital game-based learning, wherein the learning content is transmitted based on a game or simulation under the usage of digital media. For example, this is realized through serious games (SG) [9]. In this, the educational goal comes before the entertainment goal [10]. Additionally, it should be mentioned that the combination of entertainment and learning was present even before and without the use of technology [12].

Serious games have drawn the interest of the health sciences because of their interactive as well as entertaining properties [13]. Breuer and Schmitt [12] formulated 3 fields of application to discuss the effectiveness and significance of SG: preventive health promotion, support of healing processes, and the education of qualified personnel. The results seem positive at first, but there is some caution to be taken due to the wide range of SG and the varying degrees of quality, as well as the missing research on long-term effects. Furthermore, the effectiveness is dependent on the set goal [12]. Nonetheless, SG appear to bring along some advantages when it comes to learning [13]. Graafland et al [14] and Wang et al [15] have made some reviews in this regard.

Some SG make use of virtual reality (VR). This is to be understood as interactive models of reality that are simulated by computer technology. An interactive 3-dimensional (3D) gaming environment is created and becomes perceptible with the appropriate technological equipment (VR glasses, computer, smartphone) [15]. Hellriegel and Čubela [16] see the potential learning success of VR in the field of education. However, it is important to note that the usage of these technologies should be incorporated meaningfully into the lessons [16]. Barré et al [17] investigated the effects that learning a new technique with VR had on novice surgeons. They observed an improvement in the workload of the test persons. Schlegel and Weber [18] tested a nursing education class in VR and particularly emphasized the enjoyment of the students.

It is crucial to analyze the processes of technology acceptance to understand the factors of usability and acceptance. Bracq et al [19] investigated the acceptance and usability of a VR simulation for training purposes of scrub nurses. Results showed that it was accepted and suitable for vocational training.

The objective of this study is to investigate the acceptance behavior of the nursing students towards VR applications. Our research questions are: (1) To what extent does VR support learning in nursing education? (2) What factors influence acceptance and use of VR applications in nursing education? (3) How does the individual’s technical affinity influence the acceptance of VR applications? (4) In what way is the motivation to learn influenced?

## Methods

### Overview

The aim of the study was to examine the acceptability of VR applications among nursing students in the context of teaching and practicing human anatomy.

Qualitative, semistructured, and open interviews based on the unified theory of acceptance and use of technology (UTAUT2) were conducted. The theory helps to understand and depict the driving factors for usability and acceptance of technology [20]. After extension, the unified theory established 7 key constructs that influence technology use [21]. It defined performance expectancy as the degree to which using a technology will provide benefits to users in performing certain activities. Effort expectancy was defined as the degree of ease associated with consumers’ use of technology [21]. The extent to which consumers perceive that family members believe they should use a particular technology was defined as social influence. Facilitating conditions relate to the consumers' perceptions of the resources and support available to implement a behavior [21]. The extended UTAUT2 model includes 3 more determinants: (1) hedonic motivation, which is defined as the fun or pleasure derived from using a technology, (2) the price value, which is defined as the consumers’ cognitive trade-off between the perceived benefits of the technology use and the monetary cost, and (3) the habit, which is defined as the extent to which people tend to perform behaviors automatically because of learning [21].

The price value and habit determinants were not included in the qualitative interviews because monetary costs for the application as well as the habit were not relevant for this study, as there were no interviews with decision makers relating to costs. The main focus was evaluating the circumstances of acceptability of the application.

**Figure 1:**
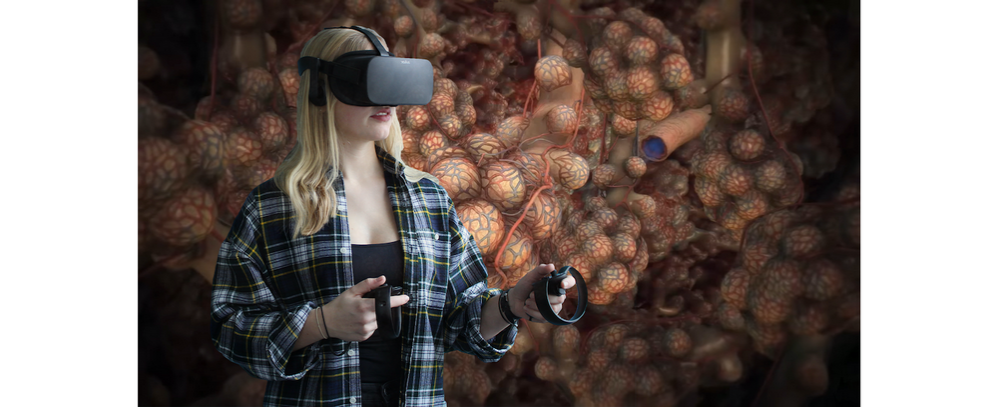
Self-created picture inside a lung.

The VR application that was worked with is a realistic 3D simulation of the human body and its anatomy. It is intended to visualize organs and their natural functions in both a healthy and sick condition (Figure 1). Physiology and state of disease can be simulated. Due to data protection reasons, the name of the application will not be mentioned.

Participants had free access to the testing of the application for the circumstances of the trial.

**Figure 2:**
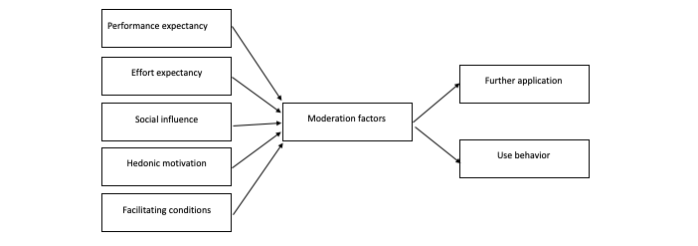
Modified unified theory of acceptance and use of technology.

Figure 2 shows the UTAUT categories in detail. In a semistructured interview (Table 1), open questions regarding performance expectancy, effort expectancy, social influence, facilitating conditions, hedonic motivation, and recommended actions and circumstances of reutilization of the VR application were asked. The questions were formulated openly so that participants could share their experiences and opinions in a storytelling way.

**Table 1:** Categories classified.

Classification Category	Definition
Performance expectancy	The extent to which the use of a technology benefits consumers in carrying out certain activities (educational or professional)
Effort expectancy	The extent of ease associated with the use of an information system
Social influence	The extent to which an individual perceives that key stakeholders believe they should use the new technology
Hedonic motivation	The extent to which the use of a technology provides fun or enjoyment
Facilitating conditions	The extent to which consumers are aware of the resources and support available to implement a scheme
Sociodemographic facts	The extent to which gender, age, and culture influence the use of the technology

Quantitative variables were assessed through a questionnaire, which included sociodemographic information (Table 2) and the *Technikaffinität erfassen–der Fragebogen* (TA-EG) questionnaire in German to evaluate technological affinity of the participants. The questionnaire contains 19 items on a 5-point Likert scale (eg, statements such as “I love to own new electronic devices” or “Electronic devices lead to mental impoverishment” [22]). Participants had to state for each of the statements how well it applied to them personally by ticking off the correct number.

Table 2 shows the sociodemographic data (age, gender, education, educational institute, experiences with VR) assessed through the questionnaire.

**Table 2:** Sociodemographic data.

Characteristics		Participants, n (%)
**Gender**		
	Male	4 (33)
	Female	8 (67)
**Age**		
	20-21	7 (58)
	22-23	4 (33)
	23+	1 (8)
**Education**		
	General A-levels	8 (67)
	Advanced technical college entrance qualification	1 (8)
	Intermediate maturity level	3 (13)
**Educational institute**		
	Nursing school	11 (92)
	University of applied science	1 (8)
**Experience with VR** ^a^		
	Yes	7 (58)
	No	5 (42)

^a^VR: virtual reality.

### Procedure

Before starting the recruitment of participants, a request was submitted to an ethics committee, which was approved soon after. The recruitment of participants took place from November 2019 to January 2020. Leaflets and flyers were placed in health care facilities (2 hospitals, 3 nursing schools). Additionally, teachers in medical nursing schools in North Rhine-Westphalia were contacted to recruit nursing students for participation. After first contact via email, potential participants who met the eligibility criteria of being in nursing education got an appointment confirmation. Nursing education was defined as training in general health care, therefore including curative care, geriatric care, and pediatric care. Because students need knowledge of anatomy in all of these health care fields, there were no specific demarcations. Students of all genders and ages were included.

Before the VR simulator training of the participants, they were asked to complete the questionnaire with sociodemographic data as well as the TA-EG questionnaire, which both were anonymized.

Afterwards, the participants got instructions on how to handle the VR simulator and went through an introduction and a short training to get familiar with the application. Participants had approximately 20 minutes to use the VR simulator.

The short training included the participants in the exploration of the functions and visualization of the lungs and the heart. They had the opportunity to customize physiology of both organs and adjust severity of specific diseases (eg, chronic obstructive pulmonary disease or arteriosclerosis).

The qualitative interview followed. During the interviews, only the participant and 2 interviewers were present, one interviewer giving the short training through the VR simulator and the other one conducting the interview.

The interviews and trainings were carried out in rotation by all of the authors, who are university students. Interviews were only conducted after the participants signed a declaration of consent for audiorecording of the interview. Duration of the interview itself was mostly about 20 minutes.

In total, 12 nursing students went through the VR training and the interview, with no one dropping out or refusing to participate. Sociodemographic data of the participants are presented in Table 2. The majority of participants were female and graduated with general A-levels. Participants overall were young, including only 1 person older than 23 years. Nearly half of the participants already had experiences with VR, mostly through conventions or exhibitions.

### Analysis

Qualitative analysis was completed using the concept maps method of Mayring, a narrative research design [23]. The method is used to summarize the essential content by first paraphrasing the raw input, then selecting, bundling, and lastly, constructing and integrating the content [23]. Audiorecordings of the interviews were transcribed and analyzed within the scope of the 5 categories included in the interviews using the Mayring method. Therefore, a structured overview of the participants’ statements is given in the “Results” regarding every category. Transcripts were not returned to participants.

## Results

### Performance Expectancy

Nursing students considered the use of VR in nursing education to be clearly beneficial. The aspect of perceived quality improvement of the learning process in anatomy was of particular importance. The perceived benefits that resulted from the contrast of digital visual learning with VR and analog learning with graphics from books were first, the time saved in the learning process, and second, the visualization of the content:

I would still learn the content with the book. Then I would go into VR and deepen what I had learned. Explore connections, for example.

The use of VR was particularly relevant in the context of the deepening of learning content. The realistic representation of anatomy content was especially emphasized, which was perceived to be a close match to real organ anatomy:

I could imagine the anatomy much better through the VR application. I really didn't think so before. It all looked so freaking real. Like I was really inside the organ.

However, it is true that VR applications tend to deepen the content already learned rather than teach completely new content. The exchange among student nurses in self-study with the help of the VR application was evaluated as homogenously positive. Consequently, self-study before exams is of great importance. Thus, students who were not able to fully learn the content in class have simplified access to independent learning with VR.

### Effort Expectancy

The use of VR applications tends to be perceived as manageable. The acquisition of VR technologies for the respective institution in which the students were located was perceived as cost intensive. Occasionally, fears of excessive demands and incorrect operation have corresponding consequences for health, manifesting themselves in side effects such as nausea, which can limit the use of VR applications. There was a shared attitude among the interviewees that, after a short introduction and testing of the VR application, the handling was no obstacle:

Before first use it sounds very complicated, but after a few minutes of use it looks easier than expected.…I immediately found my way around.

As a further aspect of the expected effort, the integration of VR into the learning process of the students makes one thing clear: An unrestricted access to the tested VR application increases the motivation to deal with necessary anatomy content. Although it also makes a change in the learning routines necessary, this was perceived as a manageable effort.

### Social Influence

The family is one of the central points of reference regarding the use and recognition of VR applications in anatomy. The opinions within the circle of friends seem to be equally relevant. However, students also expressed concerns about the experience of their teachers. The participants assumed VR applications are not yet sufficiently accepted among teachers. All in all, positive attitudes towards VR applications in the social environment increased the acceptance to use these applications oneself because social contacts often have an influence on one's own opinion making:

If you often hear about such things from friends, you are more aware of these kinds of new technologies. For me it is important to know what kind of experience they have. Especially when something is completely new to me.

### Facilitating Conditions

Facilitating conditions for the use of VR applications were perceived on different levels. These included, besides the assessment of general knowledge about VR technology, aspects of technical support, technical introduction, and cost absorption:

In my opinion, you need rooms that are already suitable for VR applications. I'm not sure if we have rooms like this in our university. And the teachers need to have time to deal with that first.

An introduction to the technology is a basic requirement for the interviewees for the assessment and subsequent use in the individual learning process. Help systems and empowerment are also explicitly called for to break down barriers of use. For the use and introduction of the VR application, trained personnel are required. The costs incurred in the acquisition would have to be fully clarified. The potential of VR applications can only be fully exploited by the students through implementation into the training curriculum of the institution:

The full benefit potential can only be exploited if learning with VR is embedded in the curriculum.

### Hedonic Motivation

The VR application promoted the motivation to learn through its playful nature, even though it was not perceived as a game application but as a support for the learning process in anatomy. The time required for learning plays an important role for the students in terms of anatomy. Through self-directed learning of new content with the VR application, learning appears to be less time-consuming, further promoting the motivation to learn in general:

Studying is stressful. It's good to have a fun alternative to normal learning. This supports me in learning, drives me forward.…Time flies in the VR.

### Moderation Factors

Overall, there were differences between the perception of the value of technology orientation and the associated knowledge about technology and the effort expectancy. The lower the average technology orientation, the more likely attitudes and opinions about the operating effort were discussed as critical. This is where the reference to social influences can be made. The more technology aware the user is, the more independent they are from social influences:

Of course I am interested in technology, it is part of my everyday life. So, I form my own opinion. About what makes sense. Especially when it comes to individual things like learning.

Furthermore, when assessing the effort required to use the VR application, it seems that it is not only the individual assessment of personal handling that plays a decisive role. The interview partners also considered the perspective of the teachers involved in the learning process. The introduction and use of the VR application should not mean any additional burden for teachers and should be easily integrated into the teaching process. This opinion of the interviewees referred mainly to the older generations of teachers:

If I were a teacher, I would at first be skeptical about the additional work. It is crucial that VR is integrated into their own training. Technical support may also be needed.

### Further Application Possibilities

In addition to anatomy, the VR application offers potential for further applications. For example, the interviewees mentioned the area of patient education, as well as behavior in an emergency. Both are central teaching topics in the training as a nurse and can be tested more intensively through real experience with a VR application. Nevertheless, a VR application is only a supporting measure to the previous learning processes and cannot completely replace them:

VR can by no means replace normal learning situations, but it can complement learning. The link between the two worlds is important. In one moment, I hear what the teacher is saying and in the other moment I can watch it live in VR. The connection between theory and practice can be solved very well.

An overview of participants’ perceptions is found in Table 3.

**Table 3:** Results summary.

Classification Category	Hypothesis
Performance expectancy	Learning is more understandable than with analog literature. Due to the better visualization, the user can memorize the content more profoundly. VR^a^ applications are able to consolidate content learned, and content can be memorized better. Due to the realistic representation of content, it can be learned better.
Effort expectancy	Side effects such as nausea can limit use. The use of the VR applications is intuitive and therefore easy to learn.
Social influence	Positive opinions towards VR applications in the social environment increase the acceptance to use them.
Facilitating conditions	If VR applications are an integral part of the training curriculum, their potential can be fully exploited. The use requires trained staff.
Hedonic motivation	The VR application promotes the motivation to learn through its playful nature. Through self-directed learning of new content, learning seems less time-consuming. Self-directed learning promotes motivation.
Moderation factors	The more technical experience the user has, the more independent they are from social influences. Previous experience is essential for optimal use. In order to integrate VR applications into nursing education, older generations must be familiarized with the applications.
Further application possibilities	Patient education and behavior in an emergency

^a^VR: virtual reality.

## Discussion

### Principal Results

The collected results presented in this paper provide new insights on the influence that VR applications have on training and further education of nursing staff. In general, the conducted study shows that the overall perception of the interviewees was quite positive towards the new technologies. VR programs can be an important, supporting part of the training to deepen learning content like anatomy. These results were consistent with previous studies on this topic [24-28].

The data show how different influencing factors, such as performance expectancy, effort expectancy, social influence, facilitating conditions, hedonic motivation, and moderating factors, can influence potential users’ acceptance of VR in anatomy education.

In this case, personal attitude towards technology in general plays an important role as well. Persons with an affinity for technology therefore consider VR programs to be more useful than persons with less affinity for technology. As a result, in the training of nursing staff, the use of VR depends on the individual characteristics of each person. In practice, this means that for optimal use of such technologies, they would have to be used in a long-term and practical way. This would ensure that every trainee could benefit from the VR application regardless of their technical affinity. Furthermore, the technology has to be easy to use and understandable for the trainees. For this reason, the interviewees stated that VR applications should be included in the curriculum. This could increase the interest of the trainees and create more technical affinity, and it would also lead to greater acceptance of VR. These statements correspond with those from previous studies, according to which a national curriculum would have a positive effect on the development of VR applications and serious games [24,29].

As already found in a study by Patterson et al [25], the results of this study show that learning with such technologies can increase the motivation of the trainees. In addition, individual and free learning is evaluated as very positive because every trainee can learn individually. However, the success of learning also depends on the personal attitude towards learning [25].

It is also mentioned in this study that social influence has an impact on the acceptance of VR technologies. It was reported that there are reservations caused by the rejection of VR technology by the family environment. However, the respondents also stated that VR might be rejected by teachers. Teachers would need to become enthusiastic about this technology in order to use VR optimally in their lessons. As Keskitalo and Ruokamo [30] described in their study, a pedagogical model must be introduced that “ensures that a more holistic and meaningful approach to teaching and learning is adopted.” This model could be used to meet the needs of the trainees and teachers to integrate simulation-based learning into the classroom. Furthermore, Keskitalo [31] stated that the beneficial parts of VR used in education depend on “the effort of the teachers to familiarize with the environment and a strong expertise in the subject, planning and flexibility.”

Due to the practicability, the interviewees mentioned that the cost factor plays a major role in the introduction of such technologies. The interviewees assume from their own findings that educational institutions often lack financial resources, which leads to the use of traditional media (eg, textbooks, presentations, videos). In addition, on the pedagogical level, the interviewed persons criticized the fact that whole classes have to learn with VR in one teaching unit. In this case, each student cannot be addressed individually, nor would the time frame be sufficient. In contrast to other studies, these statements show that further models for embedding VR applications in everyday learning have to be developed [30-32]. Furthermore, both the functionality and limitations of the VR program used in the study were demonstrated by the study participants. The visualization of the anatomical content and the mass of information were considered to be limited, and the study participants were unable to adapt all learning content to the VR application. Manufacturers of VR applications must therefore adapt their programs to the wishes of the customers and further develop the functions. Shorey et al [27] also concluded that content developers should create the VR applications in close consultation with users. In this way, subject-related content and user expertise can be integrated into the programming [27].

Overall, the interviewed persons saw great advantages in teaching anatomy with VR technologies compared with the purely analog method of learning. In addition, if optimized to its full potential, the VR technology could become a useful tool to create valuable learning environments for less motivated trainees or trainees with learning disabilities.

### Limitations

The study has various limitations, which result from the number of study participants and the qualitative research method.

Although no confounding variables were apparent during the study, the qualitative data collected are only sufficient for an insight into the topic. In order to draw further conclusions, further studies on this topic have to be conducted with a larger number of participants.

Despite the interview guidelines, we cannot exclude the possibility that questions were interpreted differently by the interview partners. Thus, statements can have different meanings. Furthermore, it is also possible that participants with a high affinity for technology were contacted in the first place. This group of people would have entered the study with different expectations than people with low affinity for technology. As a result, there might be a social bias in the study.

The study was carried out with German nursing students, so their statements refer to the German health educational system. However, the results can be applied to other health educational systems.

### Conclusion

VR is one opportunity to support learning in nursing schools. Anatomy learning content in particular can be better explored through visualization. During the study, the acceptance of the VR application was high. This shows that from the students’ point of view, the new technologies have a good chance to be introduced into nursing schools. As in other studies, the practicability and the interest of trainees was also recognized in this study [26,31,33-35]. The results reflect previously gained insights into the connection between learning and fun and the creation of new knowledge through VR, as was stated before [34]. The recommendation of the interview partners is a firm integration into the curriculum. Through integration, VR could be offered as a supporting aid that contributes to a better visualization of learning material. Thus, the pedagogically meaningful use of VR applications would be made possible.

From this pedagogical point of view, the use of VR also offers great advantages. As Bruce and Gerber [35] have mentioned, trainees perceive learning differently. The focus is on the transfer of knowledge by a teacher and practical experiences that the trainees make themselves. These skills and knowledge are then specialized by each individual for later professional challenges [36,37]. Through the practical integration of anatomical learning content, a better knowledge transfer can take place. The trainees have the chance to react to gaps in their knowledge and to repeat learning material. Furthermore, the visualization leads to a better idea of upcoming scenarios in the profession and a practice-oriented training.

However, not only nursing schools can benefit from this technology. The interview partners also saw great potential in other application areas of VR technology. Among these are the learning of practical content with virtual patients, presurgical education, or practice with patient communication. This potential has already been recognized by various studies that give information about how VR can support the learning of communication [26,32,37-39].

In view of this, the possibilities of practical training for nursing students are enormous. Due to the wide range of possible applications, nursing schools can respond to the growing challenges in the health care sector. Nursing students can thus be better sensitized to challenges and receive a further benefit in their training.

The study and the existing literature give a good outlook on what could be achieved in the health care sector through new technologies [9,12,14,15,19]. It is of great importance that actors in the health care system are familiarized with the new technologies. On the part of students and trainees, there would likely be great acceptance of VR learning content, so it is up to them to support the introduction of VR. The recommendations for action would be a discussion of the benefits of the new technologies and the embedding of the technologies in a fixed curriculum in learning institutions. Furthermore, further studies on the topic would have to be carried out to improve the evidence regarding the acceptance of VR applications and the benefit of these programs.

Even if VR technologies are still viewed skeptically from some sides, they can lead to an increase in the quality of education. The results of this study show that all participants, even those with initial skepticism, rated the VR program as positive. These statements give reason to believe that modern learning content and teaching methods are desired and demanded by the trainees.

## Appendix

Multimedia Appendix 1 CONSORT-EHEALTH (V 1.6.1) checklist.

## Abbreviations

3D: 3-dimensional
SG: serious games
TA-EG: Technikaffinität erfassen–der Fragebogen
UTAUT2: unified theory of acceptance and use of technology
VR: virtual reality
